# Influence of germinated brown rice‐based flour modified by MAse on type 2 diabetic mice and HepG2 cell cytotoxic capacity

**DOI:** 10.1002/fsn3.2043

**Published:** 2020-11-29

**Authors:** Ngoc Thi Le Nguyen, Binh Duong Thanh Nguyen, Trang Thi Xuan Dai, Son Hong Co, Thao Thi Do, Anh Ngoc Tong Thi, Ibitoye Joshua Oladapo, Ha Nguyen Cong

**Affiliations:** ^1^ Food Technology Department College of Agriculture Can Tho City Vietnam; ^2^ Department of Biology College of Natural Science Can Tho City Vietnam; ^3^ National Agro – Forestry – Fishery Quality Assurance Department Can Tho City Vietnam; ^4^ Institute of Biotechnology Vietnam Academy of Science and Technology (VAST) Hanoi Vietnam

**Keywords:** bioactive compounds, cytotoxic activity, germinated brown rice, maltogenic amylase, modified starch, starch digestion

## Abstract

This study aimed to discover whether using maltogenic amylase (MAse) to modify starch in germinated brown rice flour may enhance slow digestion starch and release more bioactive compounds (BCs) content. To achieve this aim, the starch was modified with four levels of MAse (0 U, 133 U, 266 U and 399 U MAse/g flour) for 1 hr at pH 5 and then spray‐dried to make modified flour. The biochemical impacts of the products were then accessed in normal and type 2 diabetic mice for 4 weeks. The result showed that when the starch was modified by MAse 266 U/g, a significant reduction of rapidly digested starch to 22.35% from 61.56%, an increase in slowly digested starch to 33.09% while resistant starch as 2.92% corresponding to the increase of γ‐amino butyric acid to 528.1 ± 44.1 mg/L and 120.6 ± 10.9 mg/L of ferulic acid. The extract from modified flour showed very strong cytotoxic activity against HepG2 cell (>80% inhibition). The result in vivo showed that the type‐2 diabetic mice fed with this modified product could better improve the stability of the glycemic index. Also, atherosclerotic plaque assessment further supports these findings. The results indicated that BCs released considerably couple with the changes in starch properties caused by MAse enhanced the effectiveness of this product to diabetes as well as positive effect on cytotoxic activity against HepG2 cell.

## Graphical abstract

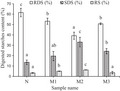

## 1 INTRODUCTION

Rice (*Oryza sativa* L.) is one of the most important cereal grains and staple food for over half of the world's population (Ghosh et al., 2019). However, under the impacts of climate change, the world's agriculture has faced a serious decline within this century and individual developing countries face even larger declines (Bhattacharya, 2019). Many previous studies have shown that the continuous intake of germinated brown rice (GBR) helps to prevent diabetes in healthy human subjects (Ren et al., 2020) and free‐living patients with impaired fasting glucose or type 2 diabetes (Hsu et al., 2008). As a result, GBR is known as a functional food with plenteously bioactive components, which increased significantly including γ‐butyric acid (GABA), ferulic acid (FA), γ‐oryzanol (GORZ), and several phenolic acids (Cho & Lim, 2016) with a positive effect on human health and positive cytotoxic activity against human hepatocellular adenocarcinoma cell (HepG2 cell) (Imam & Ismail, 2013). According to Balasubashini et al. (2003), FA had a beneficial effect in lowering the blood pressure in streptozotocin‐induced diabetic rats. Besides, GABA was reported to modulate the immune response by inhibiting pro‐inflammatory CD4 + T‐cell responses, modulating the cytotoxicity of CD8 + T cells in vitro and inhibiting cell autoimmunity and inflammatory responses in a mouse model of type‐1 diabetes (Tian et al., 2004). Usuki et al. (2008) also found that the acylated steryl glucoside (ASG) in GBR can help to control blood sugar levels in diabetic patients by increasing the levels of beneficial enzymes. As suggested by Roohinejad et al. (2010), the antihypercholesterolemic effect of GORZ is partial due to its sterol moiety, which is partly splitted off from the FA part in the small intestine by cholesterol esterase. Some studies about the development of various food products from GBR are carried out, such as GBR‐based flour, yogurt‐like product (Cáceres et al., 2019; Lee et al., 2019). However, there is still no report about the effects of the product on diabetes and HepG2 cell from these studies. GBR is considered a gluten‐free functional food owing to its good digestion and absorption properties (Lee et al., 2019). Although the germination process reduced sugar, as we all know that, starch is still the major component that accounts for 77.7% in GBR (dry weight basis) **(**Moongngarm, 2011). The long‐term consumption of fast digestible products may contribute to promote human diseases such as type 2 diabetes, cardiovascular disease, and obesity (Lovegrove et al., 2017). Therefore, a new treatment which is modified starches has emerged in order to obtain healthier carbohydrates such as slowly digestible starches (SDS), resistant starch (RS) and rapidly digestible starches (RDS) through the applications of different enzymes and techniques—physical or chemical modification (Román et al., 2017). In order to process functional products from GBR, studies on increasing the rate of resistant starch from GBR are needed. This is the basis for bioactive compounds to take effect in processed products.

Enzymatic modification is the most desirable and green method since it possesses higher substrate selectivity and product specificity, fewer harmful by‐products (Román et al., 2017). Among several enzymes, maltogenic amylase (MAses) (EC 3.2.1.133) has gained so much attention due to their industrial applications, including baking industry and starch conversion applications as anti‐staling agent for increasing shelf life, synthesis of noncarcinogenic sweeteners, synthesis of novel carbohydrates, and the development of new drugs for the treatment of obesity, hyperlipidemia, dental caries, and diabetes, as well as changing its rheological properties and enhancing its oxidative stability (Bae et al., 2002; Haghighat‐Kharazi et al., 2020). Several authors have reported that using MAse in order to slow down the digestion of starches or flours are via the catalytic mechanism including (i) increasing the ratio of short chains; (ii) increasing amounts of α,1–6 linkages for smaller chains and a higher branching which helps to slow down the digestion of starches; (iii) increasing prebiotic isomaltooligosaccharide contents (Ao et al., 2007; Román et al., 2017). Therefore, this study investigated the effects of MAse on MGBRF and BCs release with possible health benefits on normal and diabetic mice fed with the diet as well as on cytotoxic capacity on HepG2 cell.

## 2 MATERIALS AND METHODS

### 2.1 Chemicals

The GABA, FA, GORZ, enzymes such as MAse (Amylase, Maltogenic from *Bacillus* sp.; declared activity ≥ 10,000 MANU/g product; code A2986), Invertase (Invertase from baker's yeast (*S*. *cerevisiae*)‐Grade VII; enzyme activity ≥ 300 units/mg product; code I4504), Arabinose (product number W325501), and 1,1‐diphenyl‐2‐picrylhydrazyl (DPPH) standards were obtained from Sigma‐Aldrich Chemical. All the solvents used in the experiments, 4‐dimethyl aminoazobenzene‐4‐sulfonyl chloride, 5‐sulfosalicylic acid dihydrate are analytical reagents, or HPLC grade and were purchased from Merck, Fisher and J.T.Baker‐Mallinckrodt Baker Chemical. At once, glucoseamylase (Attenuzyme® Core, glucan 1,4‐alpha‐glucosidase from *Aspergillus niger*; declared activity 1,600 AGU/g liquid), alpha‐amylase (*Termamyl*® 120 L from a genetically‐modified strain of *Bacillus licheniformis)* of Novozymes (Novozymes A/S). Sulforhodamine B (SRB) from Sigma and other chemicals with purity level for the study were provided by CEMACO joint‐stock company.

### 2.2 Preparation of GBR nonmodified flour

The paddy rice IR 50,404 variety was received in Cuu Long Delta Rice Research Institute in Can Tho city, Vietnam and stored at the temperature of 20 ± 2°C) and dehusked (Mini Paddy Husker, model STHU‐35S, Satake) to brown rice; then, the rice seeds were soaked in a buffer solution with pH 4 (Na_2_HPO_4_.12H_2_O and citric acid) and 0.6% glutamic acid and incubated in an incubator (SANYO MCO‐5AC Incubator) which had a maximum yield of 0.5 kg/batch at a temperature of 37°C with 24 hr incubation period. Thereafter, the GBR was dried at 60°C for 4 hr and milled into flour, packaged and stored at −18°C for use in the experiments.

### 2.3 Modification of starch in GBR using MAse and making MGBRF

The GBR‐based flour was weighed in Erlen and buffer of pH = 5 in each flask (0.1 M Disodium hydrogen phosphate dodecahydrate; 0.1 M Citric acid monohydrate), was added to achieve a mixture of 20% substrate, and then stirred well. The sample flasks were heated to 80–85⁰C in a boiling water tank with 90–95⁰C temperature to gelatinize the starch. After 20 min, the samples were cooled down and incubated in the water bath set at 60⁰C. The addition of enzymes to the corresponding labeled Erlen with 0 U, 133 U, 266 U, and 399 U MAse/g dry weight of GBR‐based flour, respectively, was performed after having a thermal balance between inside and outside. The incubation times were 0 and 1 hr. Subsequently, the samples were filtered through a curtain cloth to get the soluble components and to remove residue before analysis, then stored at −18⁰C for further analysis of BCs and digestibility indexes of starch. The remaining samples were dried by Spray Dryers (LabPlant‐SD 06, UK) to get MGBRF and then stored at −21°C in the freezer, Sanaky, Vietnam.

### 2.4 Effect of MGBRF extract on cytotoxic activity against HepG2 cell

The cytotoxic activity against HepG2 cell was done following the method of Skekan et al. (1990). Briefly, the MGBRF was extracted with two ratios with water as 1:5 and 1:10 (w/v) at 60°C for 15 min. After that the sample was mixed and centrifuged at 16,000 g. The supernatant was filtrated through 0.2 μm membrane before treated with HepG2 cell. In order to test the cytotoxic activity against HepG2 cell, 40 µl of sample was mixed with 60 µl of DI water (C1), diluted to 5 times (C2) (=C1/5), C3(=C2/5) and C4 (=C3/5) and then put all samples into wells of 96‐well plates (Corning, USA) containing HepG2 cell (6,000 cell/well). The mixture was incubated for 48 hr by ELISA Plate Reader (Bio‐Rad). After 48 hr, the cells were fixed by TCA for 1 hr to stop reaction, stained with sulforhodamine B (SRB) for 30 min at 37°C, washed 3 times with acetic acid, and allowed to dry at room temperature. Read OD results at 515–540 nm on ELISA Plate Reader (Bio‐Rad). The percentage inhibiting cell growth in the presence of the test substance was determined. For control, TBUT (180 µl) was used as the control for day 0. Ellipticine was used as a positive control with 4 levels (10 μg/ml, 2 μg/ml, 0.4 μg/ml and 0.08 μg/ml). 10% DMSO is always used as a negative control. The sample determined as a positive effect on cytotoxic activity against HepG2 cell when the inhibition level was higher than 50% of inhibition of HepG2 cell.

### 2.5 Extraction and analysis of GABA by HPLC

The GABA was separated and analyzed by the method of Banchuen et al. (2010) with slight modifications. Adding 1 ml of liquid samples to 9 ml of deionized water in Erlen, then shook for 90 min at room temperature. Thereafter, 1 ml of 3% (by volume) sulfosalicylic acid was added, and the mixtures were centrifuged by Hermle Labotechnik Z323K Centrifuge at 6,000 g for 10 min. Adding 100 μl of the supernatants, 100 μl of 100 mM NaHCO_3_ and 100 μl of 4 mM 4‐dimethylaminoazobenzene‐4‐sulfonyl chloride (was dissolved with acetonitrile solvent) to Eppendorf, then vortexed. The mixture was heated at 70°C for 20 min to derivatization effect, after that, it was cooled down and added in 0.5 ml of absolute ethanol on ice and centrifuged at 16,000 g and 4°C for 5 min. Finally, the supernatants were analyzed on HPLC (Shimadzu), with Ascentis® C18 HPLC column, 25 cm × 4.6 mm, 5 µm (Supelco). The HPLC was equipped with a UV‐Vis detector (SPD‐20A, Shimadzu) set at 465 nm wavelength. The mobile phases were 25 mM ammonium acetate buffer containing 0.1% acetic acid and acetonitrile (26:74) adjusted at the flow rate of 1 ml/min with 10 µl of injection volume and temperature column oven at 55°C. Pure GABA was used as a calibration standard.

### 2.6 Extraction and analysis of FA by HPLC

Total FA content was determined using high‐performance liquid chromatography as described previously (Banchuen et al., 2010). The samples (3 ml of liquid) were extracted with 10 ml of 1 M NaOH and shaken for 3 hr at room condition then neutralized by 5 ml of 2 M HCl. The mixture was extracted three times with 10 ml of ethyl acetate, each time for 5 min. Subsequently, the supernatant was evaporated at 40°C in vacuo to remove the ethyl acetate. The residue was dissolved in 3 ml of MeOH:DI (v/v = 1:1) and subjected to analysis on the Shimadzu HPLC system equipped with a UV‐Vis detector on an Ascentis® C18 HPLC column, 25 cm x 4.6 mm, 5 µm (Supelco) and was detected at the wavelength of 320 nm and temperature of 40°C. The mobile phases were acetic acid (2.5% by volume) and acetonitrile (32:68) at a flow rate of 1 ml/min. Pure FA was used as a standard for calibration.

### 2.7 Extraction and analysis of GORZ by HPLC

GORZ content was determined to follow the method of Cho et al. (2012). 1 ml of the sample was extracted in acetone: ethanol (contain 0.1% of Butylated Hydroxy Toluene or BHT) with ratio 9:1 (volume by volume). The mixtures were shaken for 90 min at room temperature and centrifuged at 6,000 g in 4⁰C for 5 min. The supernatants were collected and the residues were extracted two more times and analyzed on the HPLC (Shimadzu), with an Ascentis® C18 HPLC column, 25 cm × 4.6 mm, 5 µm (Supelco). The HPLC was operated with a UV‐Vis detector set at 330 nm wavelength and a temperature column oven of 35°C. The mobile phases were methanol: acetonitrile: dichloromethane: acetic acid (50:44: 3:3) and the flow rate of 1 ml/min. γ‐Oryzanol was used as a standard for calibration.

### 2.8 Determination of reducing sugar content

The reducing sugar content was determined by the method of Cho et al. (2000). The hydrolysate was filtered through filter paper. 1 ml of filtrate was taken and added in 3 ml of DNS reagent and heated up for 5 min in boil water. The color absorption was determined by the Multiskan Spectrum at 540 nm. Pure glucose was used to calculate for a standard line.

### 2.9 Determination of RDS, SDS, and RS fractions of MGBRF in vitro

For nutritional purposes, starch is divided into rapidly digestible starch (RDS), slowly digestible starch (SDS) and resistant starch (RS) based on the rate and extent of its digestion in the in vitro Englyst assay (Englyst et al., 1992), with some modifications. The enzyme solution was prepared immediately before use (weighed 3 g pancreatin into each of six screw‐cap centrifuge tube, added in 20 ml of distilled water, mixed, stirred for 10 min, then centrifuged at 6,000 g for 10 min). 15 ml supernatant was taken from each tube in Erlen and mixed 6 ml enzyme mixture of α‐amylase: glucoamylase ratio 1:1 ([Enzyme mixture] ≈200 AGU/ml), 4 ml invertase ([Invertase] ≈460 U/ml)). Thereafter, pipetted 1 ml of each sample into Vitro, added in 5 ml of 0.1% arabinose, and 10 ml pepsin‐gellan gum; Vitro tubes were capped, mixed thoroughly and submerged into the water bath at 37⁰C for 30 min with the addition of 5 ml (0.1 M sodium acetate). 5 ml of previously prepared enzyme solution was added, vortexed, and incubated at 37⁰C for 20 min and 120 min, later 0.4 ml of hydrolysate in a labeled tube containing 8 ml of absolute ethanol was removed, this were G20 and G120 portion, respectively. Then, the tubes were vortex‐mixed and placed in the boiling water bath for 30 min, cooled with ice‐water (0⁰C) for 15 min. Furthermore, 10 ml of 7 M KOH was added into each tube and kept for 30 min in an ice‐water bath. Continuously, 10 ml of 0.5 M acetic acid was added, vortexed, and 1 ml of this supernatant was taken into other Vitro with 1 ml of enzyme mixture added. The tubes were incubated for 30 min at 70⁰C and heated in the bath up to 100⁰C for 10 min. Finally, cooled to room temperature and diluted with 12 ml of absolute ethanol to obtain total glucose (TG). The values of G20, G120, and TG were combined to calculate RDS, SDS, and RS.

### 2.10 Detection of the glycosides BCs using TQD UPLC/MS

The starch from the GBR‐based flour was modified by optimal MAse condition of 60°C for 1 hr with pH 5 as stated above. Then, the sample was shaken, centrifuged at 6,000 g for 6 min, and filtrated through a 0.2 μm syringe before injected to TQD UPLC/MS system (Waters Corp). The mobile phase conditions of UPLC for GABA and FA were A: 100% methanol and B: water. The injection volume was 10 μl. The retention time was 5 min. The column was ACQUITY UPLC CSH C18 1.7 μm, 2.1 × 50 mm from Waters. For GORZ analysis, the column was Luna 5 μm NH_2_ 100Å 2 × 150 mm from Waters and the retention time was 6 min. Other conditions were the same as for GABA or FA. To detect whether or not the BCs modified by glucose or/and maltose, mass spectra were used to detect from the range of 50 – 300 m/z. Comparing to standard peaks of GABA and FA, all modified glycosides BCs peak appeared in the mass spectra if they exist.

### 2.11 Effect of MGBRF on Type 2 diabetic mice in vivo

42 Swiss strain and diabetic mice (IVAC, Viet Nam) with ages 6–8 week, 25–30 g ideal weight used for the experiments were bought from the Pasteur Institute of Nha Trang, Vietnam. The mice were housed in an environmentally controlled room (30–32°C, 65%–75% relative humidity) and fed ad libitum with chow diet (crude protein: 23%; crude fat: 8%; crude fiber: 4%; moisture: 10%) for healthy maintenance. Optimal samples from the experiments, investigating changes in BCs and in vivo results, were spray‐dried at 160°C (LabPlant‐*SD* 06, Labplant UK Ltd, UK), with peristaltic pump: 485 ml/h and stored at −18⁰C in PA vacuum packaging. Each sample (450 mg/kg body weight) was dissolved into 5 ml of boiling water to feed the mice. The nutritional components of samples in this experiment are shown in Table 1.

**Table 1:** The nutritional components of samples in this experiment

The components	Content (mg/g, dry basic)	
	NS (nonmodified GBR starch spray drying)	MA (modified by MAse GBR starch spray drying)
Total protein	51.50 ± 4.50	69.80 ± 1.10
Total lipid	50.30 ± 5.50	57.30 ± 1.50
Total starch	783.60 ± 6.40	784.40 ± 1.71
+ Slow digested starch (SDS)	136.00 ± 2.15	330.90 ± 4.57
+ Resistant starch (RS)	33.20 ± 3.10	62.40 ± 1.40
+ Rapidly digested starch (RDS)	615.60 ± 4.09	392.10 ± 3.43
γ‐amino butyric acid (GABA)	2.04 ± 0.02	5.28 ± 0.44
Ferulic acid (FA)	0.45 ± 0.17	1.21 ± 0.11
γ‐oryzanol (GORZ)	‐	‐
“‐,” not detected		

Note

The values in the table were averages of 3 replicates. NS: Sample control that did not use MAse, MA: GBR was modified by 5,314.01 U of MAse. NS and MA samples were used in mice experiments.

### 2.12 Glucose tolerance (GT) test in mice

Test samples included glucose (Glu), sucrose (Suc), modified (MA‐S) and nonmodified (NS) GBR base flour. Glucose tolerance (GT) test in mice was carried out using the method of Dura et al. (2016). Four mice males, 6–8 weeks’ ages, 25‐30g ideal weight, were allowed to fast for 4 hr, and the blood glucose level was defined before testing. The samples (0.2 ml) were pumped directly into the esophagus, and blood glucose was determined at 0, 15, 30, 60, and 120 min by ACCU Check Active (ROCHE).

### 2.13 Glycemic index (GI) test in type 2 diabetic mice

There were three animals per cage, and they were divided into six groups: NS (normal, noninduced type 2 diabetes, fed with nonmodified spray‐dried), MA (noninduced type 2 diabetes fed with modified diet), T2D‐NS (type 2 diabetes group fed with nonmodified), T2D‐MA (type 2 diabetes group fed with modified diet), T2D‐NS‐*M* (type 2 diabetes group fed with nonmodified spray‐dried diet and metformin), and T2D‐MA‐*M* (type 2 diabetes group fed with modified diet and metformin). The mice in the group using metformin were given hypoglycemic agents (Glucophage 500 mg Tablets, 300 mg/kg BW) and pumped 0.2 ml directly into the esophagus after 2 hr using sample test. Feeding lasted for four weeks and the result was recorded every week. The samples and experiments were administered using a 4 × 4 Latin square design. Recorded results and blood glucose units were calculated according to Joslin Diabetes Center, US website (Accessed 30th October, 2019).

### 2.14 Blood plasma biochemical and antioxidant activity analysis

Total cholesterol (TC), triglycerides (TG), High‐Density Lipoprotein Cholesterol (HDL‐C), low‐density lipoprotein cholesterol (LDL‐C), alanine aminotransferase (ALT), aspartate transaminase (AST) in blood, malondialdehyde (MDA), glutathione reductase (GSH) in liver homogenates were determined with enzymatic methods using corresponding Ams Ellipse Automated Analyzer (AMS, Italy).

### 2.15 Statistical analysis

Results are expressed as mean ± standard deviation and all experiments were conducted in triplicates. Data were calculated on average and the standard deviation by Excel 2010 software. Data were statistically analyzed for ANOVA using the Statgraphics Centurion XV software (Version 15.2.11, Statgraphic Technologies, Inc.). LSD test at a 5% significance level (*p* ≤ .05) was used to determine the significant difference between treatments.

## 3 RESULTS AND DISCUSSION

### 3.1 Enzymatic hydrolysis on GBR‐based flour

**Figure 1:**
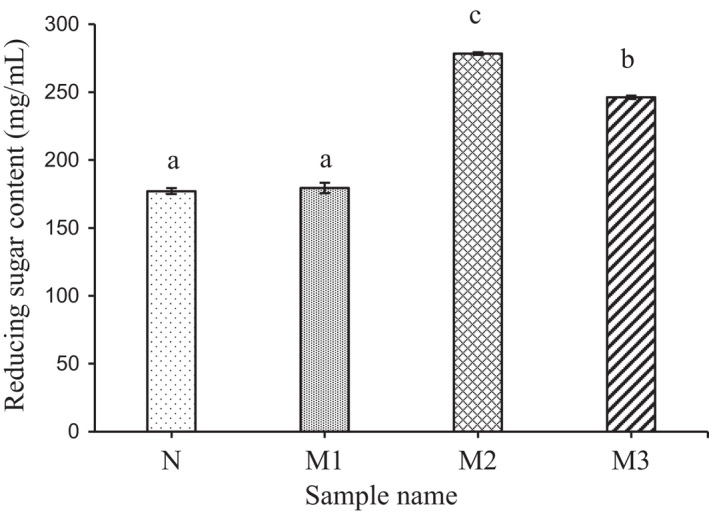
The reducing sugar content generated at difference concentration of MAse. Significant differences between values were indicated by different letters (*p* < .05). N: Sample control was not MAase, M1: GBR was modified by 133 U MAse/g dry weight of GBR‐based flour, M2: GBR was modified by 266 MAse/g dry weight of GBR‐based flour, M3: GBR was modified by 399 MAse/g dry weight of GBR‐based flour

The results in Figure 1 show that the reducing sugar content increased linearly with the MAse enzyme concentration, attained the peak of 278.42 g/L at 266 U MAse/g dry weight of GBR‐based flour, and lowered down to 246.25 g/L at the level of 399 U MAse/g dry weight of GBR‐based flour (Figure 1). The results indicated that the MAse enzyme provided the best result at 266 U MAse/g dry weight of GBR‐based flour under the conditions of this experiment. It is consistent with the kinetic theory of enzymes, as the concentration of enzymes increases, so does its catalytic activity. However, the critical threshold of the concentration continues to increase, whereas the activity decreases. Because the released hydrolysate inhibited the activity of the enzyme, the growing up in the concentration of solutes in the hydrolysis medium to reduce the enzyme flexibility or at an excessive concentration of the enzyme reserve inhibited themselves. MAses hydrolyze α‐1,4‐glucosidic linkages of starch from the nonreducing head and its derivatives to maltose reducing the chain length of the polymer. It cleaves α‐1,4 linked linear chains of amylose and amylopectin followed by transglycosyl reaction to create new α‐1,6 linked branch chains (Ao et al., 2007). MAse also exhibits high transglycosylation activity via the formation of various glycosidic linkages such as α‐1,6 producing branched oligosaccharides (Le et al., 2009). MAse enzymes hydrolyzed various carbohydrates such as starch, cyclomaltodextrins and pullulan to produce maltose, glucose, and panose. Besides, it also could transfer monosaccharides or disaccharides that were products of hydrolysis to other receptors such as D‐glucose, maltose, cellobiose, and lactose (Bae et al., 2002). According to Cho et al. (2000), one unit of enzyme activity was defined as the amount of enzyme producing the reducing sugar equivalent to one unit change. In order to improve the modification by MAse, several studies with combined enzymatic treatment on extruded maize flour (higher glucose and dextrin level were achieved when the sample treated by extruder), 300 U of branching enzyme/g flour (BE) and 7 U of maltogenic α‐amylase/g flour (MAse) were used to get higher modification level (Ao et al., 2007; Román et al., 2017). Another report of tapioca modified starch to produce amylolytically resistant starch, and the branching enzyme (1,000 U/g starch) reaction product was further treated with maltogenic amylase (1,000 U/g starch) (Le et al., 2009). It is clearly that reduced sugar is a product of MAse, it is also a material for next step of modification of the MAse to create new α‐1,6 linked branch chains. Since the result of Figure 1 indicated that reducing sugar content increased linearly with the MAse enzyme concentration, therefore, the levels of enzyme used in this study showed an effective for modification.

### 3.2 Effect of MAse on bioactive compounds and cytotoxic activity against HepG2 cell

The result in Table 2 showed that the contents of GABA and FA in the samples using enzymes were higher than the nonenzyme control sample. The content of these compounds changed with enzyme concentration and had a significant difference (*p* < .05). The components rose together by advancing MAse activity at 266 U MAse/g dry weight of GBR‐based flour, both of them had the highest level with 528.1 mg/L of GABA and 120.6 mg/L of FA but slightly decreased at the level of 399 U MAse/g dry weight of GBR‐based flour. So, when using the enzyme MAse to modify the GBR base flour showed that there was a positive impact in its ability to release functional compounds. In this study, the GBR‐based flour was used as material for modification by MASe. After enzyme reaction, the mixture was filtrated by a curtain cloth to get the soluble components as well as discard the water insoluble component or solid. So, mostly nonmodified and less‐modified starches as insoluble components were kept in this insoluble component part while released protein as well as bioactive compounds into soluble part. It was then used for spray drying to make MGBRF. As a result, there were an increase of protein and bioactive compounds in the MGBRF comparing to nonmodified spray‐dried flour as shown in Table 1. This result is consistent with studies on using hydrolyzed enzymes to break down raw materials at initial bonds, thereby helping to release other ingredients as well as functional compounds into soluble part (Hammed et al., 2013; Liu et al., 2017; Nkhata et al., 2018). A study of phenolic compounds in white rice, brown rice, and GBR reported that insoluble and soluble bound FA in GBR was 163.9 mg/kg and 2.8 mg/kg of flour, respectively. It was the major phenolic compound and the most abundant insoluble phenolic compound which existed in the form of free, soluble conjugate and insoluble bound. Most of these compounds are bound to polysaccharides containing glucose, arabinose, xylose, galactose, rhamnose, and mannose residues in the cell wall (Tian et al., 2005). In addition, GORZ, mainly composed of esters of trans‐ferulic acid (trans‐hydroxycinnamic acid) with phytosterols was not detected in all the test samples (filtrate) despite being detected in solid samples (sample tested) (Lerma‐Garcia et al., 2009). The manufacturer had information that water solubility of GABA was 103.1 g/L at 20°C; it dissolved well in water and no available data of FA and GORZ. On the other hand, Kaukovirta‐Norja et al. (2004) reported that steeping and germination of cereals could produce enzymes to break down the cell walls surrounding many compounds and that the free phenolic acids and bound phenolic acids might then be released. Furthermore, the study of Hammed et al. (2013) indicated that enzymatic hydrolysis could enhance the release of biological compounds from raw materials. Therefore, FA was discovered when the enzyme or complex enzymatic hydrolysis releases phenolics, and hydrolysis of the phenols binding with sugar, fatty acid, and protein molecules releases maximum phenolic (Liu et al., 2017). Besides, Uraji et al. (2013) succeeded in enhancing the enzymatic production of FA from defatted rice bran with the combination of three xylanases, an α‐l‐arabinofuranosidase, and an acetyl xylan esterase from *Streptomyces spp*. This enzyme combination also had an effect on FA production from other biomasses, such as raw rice bran, wheat bran, and corncob. Moreover, an enzymatic hydrolysis process for FA extraction from wheat bran was optimized gradually by enzymatic pretreatment with alcalase and termamyl, and a final enzymatic hydrolysis with pentopan and feruloyl esterase extraction yield higher than untreated condition (Ferri et al., 2020).

**Table 2:** Changes of GABA, ferulic acid, γ‐oryzanol content follow enzyme concentration in modified GBR flour by MAse

Sample	Bioactive compounds content (mg/L)		
	GABA	FA	GORZ
*N*	203.9 ± 1.8^a^	45.4 ± 1.7^a^	‐
M1	286.7 ± 4.8^b^	64.5 ± 4.1^b^	‐
M2	528.1 ± 44.1^d^	120.6 ± 10.9^d^	‐
M3	439.7 ± 31.2^c^	104.0 ± 8.1^c^	‐
“‐,” not detected			

Note

Significant differences between values in the same column were indicated by different letters (*p* < .05). *N*: Sample control was not used MAse, M1: GBR was modified by 2,657.01 U of MAse, M2: GBR was modified by 5,314.01 U of MAse, M3: GBR was modified by 7,972.02 U of MAse.

The result in Table 3 showed that there were no the glycosides BCs detected after modifying by MAse. This result indicated that BCs releasing from modification did not change any functional properties. Some studies have shown that using MAse is capable of denaturing some compounds, through new binding to another receptor, helping them to enhance their solubility or activity (Bae et al., 2002; Cho et al., 2000; Li et al., 2004). Hence, to increase some components in this study, the experimental samples were analyzed on TQD UPLC/MS and results showed that there was no any modification or change in the above functional compounds (Table 3). Therefore, the results of increasing GABA and FA contents in this study were suitable for the studies using enzymes to hydrolyze bonds in order to enhance the release of functional compounds. As for GORZ, the study has not found any effect, since it was not detected in the filtrate.

**Table 3:** Detection of the glycosides BCs after modifying by MAse using TQD UPLC/MS

	GABA	Binding of GABA with glucose	Binding of GABA with maltose	FA	Binding of FA with glucose	Binding of GABA with maltose
*N*	+	−	−	+	−	−
M2	+	−	−	+	−	−

Note

Sample control (*N*), M2: GBR was modified by 266 MAse/g dry weight of GBR‐based flour.

The result in Table 4 indicated that the extract from M sample (MA‐S) showed good inhibition of growth of HepG2 cancer cells, reaching over 50% of both modified samples, up to 81.73% at 1:5 and 68.33% at 1:10 (w/v) sample, respectively. While nondenatured flour extract samples (NS) did not show the ability to inhibit the growth of cancer cells HepG2 (Just 30.69% inhibition). The results indicated that the more diluted of the sample, the more decreased of the inhibitory activity in both samples. The positive control of ellipticine was also stable in the experiment. GBR is known as a functional food with plenteously bioactive components, which increased significantly including γ‐butyric acid (GABA), ferulic acid (FA), γ‐oryzanol (GORZ), and several phenolic acids with a positive effect on human health and positive cytotoxic activity against human hepatocellular enocarcinoma cell (HepG2 cell) (Imam & Ismail, 2013). After modification by MAse, there were no the glycosides BCs detected. Beside, an increase of such BCs caused by releasing to soluble part after modification made concentration of BCs in MGBRF product was higher than nonmodified one. It indicated that bioactive compounds in the MGBRF still have good effect on cytotoxic activity against HepG2 cell even though the flour was modified by MAse.

**Table 4:** Cytotoxic activity of GBR‐based flour extract against HepG2 cell

Sample	MA‐S		NS		Ellipticine
Diluted ratio with water (w/v)	1:5	1:10	1:5	1:10	
Percentage of inhibition on HepG 2 cell	81.730 ± 2.040	68.330 ± 2.130	30.690 ± 1.090	24.330 ± 0.620	88.110 ± 0.810

Note

MA‐S, Modified germinated brown rice flour extract; NS, Control sample (Nonmodified germinated brown rice flour).

### 3.3 In vitro starch digestibility

**Figure 2:**
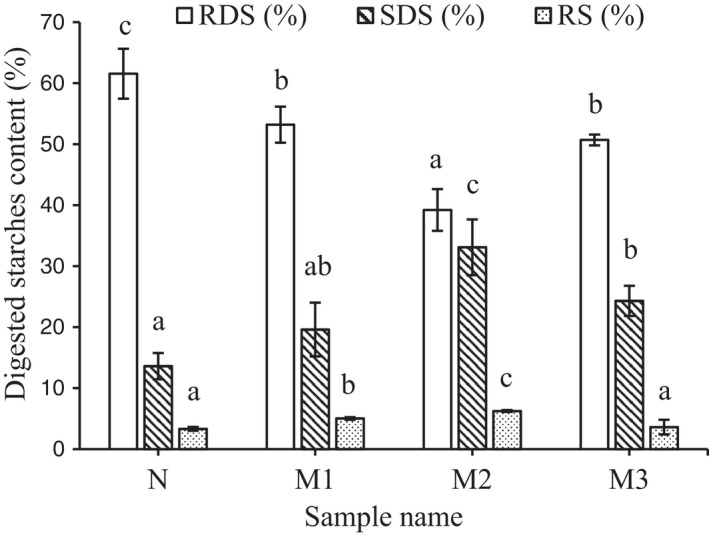
Effect of MAse concentration to ability starch digestion in vitro. Significant differences between values in the same digested starches content (RDS or SDS or RS) were indicated by different letters as a, b and c (*p* < .05). N: Sample control was not MAase, M1: GBR was modified by 133 U MAse/g dry weight of GBR‐based flour, M2: GBR was modified by 266 MAse/g dry weight of GBR‐based flour, M3: GBR was modified by 399 MAse/g dry weight of GBR‐based flour

The result in Figure 2 showed the effect of MAse concentration to ability starch digestion in vitro. It was clearly shown that modifying starch by MAse changed starch digestibility significantly in GBR‐based flour. Compare to the control sample, the GBR starch product prepared using 266 U MAse/g showed a significant reduction of rapidly digested starch by 22.35% (control sample was 61.56%) and with a concomitant slightly reduction of resistant starch by 2.92% (control sample was 3.32%). Meanwhile, the SDS content was improved greatly in all of the samples having an enzyme that jumped up from 13.60%, 19.61%, to 33.09% and went down a bit to 24.31% at 0 U, 133 U, 266 U, and 399 U MAse/g respectively.

By nutritional purposes, starch is divided into rapidly digestible starch (RDS), slowly digestible starch (SDS) and resistant starch (RS) based on the rate and extent of its digestion in the in vitro Englyst assay (Englyst et al., 1992). So far, there have been many shreds of evidence on the ability to increase the content of slowly digested starch and resistant starch of enzyme maltogenic amylase when modifying starch from different sources. Ao et al. (2007) reported that the normal maize starch subjected to MAse (7 U/g dry weight of starch) for 5 hr produced 18.8% SDS and 13.5% RS with a reduction of 19.8% RDS, which was similar to the results obtained in this study. According to Miao et al. (2014), the maltogenic amylase treated maize starch for 1 hr yielded 14.6% of SDS content which was lower to data presented in Figure 2, whereas a nearly equal in RS content. The maltogenic amylases have been used to catalyze hydrolytic and transfer reactions to form new α‐1,6 linkages for smaller chains and a higher branching which help to slow down the digestion of starches (Ao et al., 2007; Román et al., 2017). The MAse modified starch also had a greater RS which might be attributed to the branched oligosaccharide production by maltogenic amylase via transglycosylation (Román et al., 2017). In addition, the contents of RDS, SDS and RS changed to 45.9%, 46.3%, 7.8%, or 53.8%, 38%, 8.2% upon GBR hydrothermal treatments or 51.7%, 39.6%, 8.7% in GBR ultrasonicated, respectively (Chung et al., 2012; You et al., 2016). Therefore, the MGBRF helped to enhance BCs content and the reduction of rapidly digested starch, increased slowly digested and resistant starch in the in vitro digestibility test. The results were obtained also shown that the enzyme effectively worked best at 266 U/g in this study. The product was then tested in vivo on type 2 diabetic mice.

### 3.4 In vivo glucose tolerance (GT) test in mice

The results in Table 5 showed blood glucose concentrations in mice after intake of glucose, saccharose, nonmodified and enzymatic modified GBR starches. The released blood glucose after the intake of an amount of glucose (reference) reached a maximum level after 15 min of ingestion and rapidly dropped to the initial level after 120 min. Mice that took saccharose and the glucose content reached a peak after 60 min. Ingestion of enzymatically modified GBR starches lowered the level of blood glucose released as compared to nonmodified GBR starches which peaked at 30 min and then dramatically reduced for longer digestion. Meanwhile, the highest level of blood glucose was released at 120 min after the intakes of nonmodified GBR starches.

**Table 5:** Glucose content (mg/dL) of mice tested feeding the surveyed samples at different times

Sample name	Time (min)				
	0	15	30	60	120
NS	158.47 ± 15.89^bA^	203.44 ± 15.61^bB^	219.63 ± 16.94 ^bB^	232.23 ± 31.88 ^bB^	272.70 ± 65.52 ^bAB^
MA‐S	134.18 ± 14.87^aA^	183.65 ± 28.60^aB^	214.69 ± 27.49 ^aB^	189.05 ± 36.34 ^aB^	146.78 ± 30.44 ^aAB^
Glu	167.46 ± 2.08^aA^	193.55 ± 3.12^aB^	178.26 ± 4.15 ^aB^	169.26 ± 2.08 ^aB^	147.67 ± 14.54 ^aAB^
Suc	144.08 ± 4.15^abA^	204.34 ± 1.04 ^abB^	218.73 ± 5.19 ^abB^	252.91 ± 98.67 ^abB^	202.54 ± 9.35 ^abAB^

Note

The lowercase letters (a, b, c, d) in superscript indicated a statistically significant difference between treatments in each time by column (*p* < .05). The uppercase letters (A, B, C, D) in superscript indicated a statistically significant difference between the time frames of each treatment by row (*p* < .05). Glu is glucose, Suc is sucrose, NS is GBR base flour nonmodified, MA‐S is GBR base flour modified by MAse.

The model of glucose tolerance increased rapidly in the first 15 min which was similar to the report of Dura et al. (2016). Although the modified GBR starches showed the lowest blood glucose levels, they digested saccharose more which took some time to release glucose because it was needed to be hydrolyzed into simple sugars before being absorbed into the bloodstream instead of a direct way as glucose. The longer ingestion time of the nonmodified GBR starches might be due to the fine structures of amylose and amylopectin molecules of these starches which had higher degrees of crystallinity. These results were due to the high amounts of SDS and RS in the annealed starches than those in the heat‐moisture treated starches and native starches. Thus, the different results of blood glucose released after the intakes of varying samples were positively related to total amounts of SDS and RS. The high amount of SDS and RS delayed the digestion time and blood glucose‐releasing duration (Pham et al., 2015). Shin et al. (2007) reported that the much lower blood glucose level after the intake of acid‐treated rice starch compared to the native starch, presumably due to the content of SDS and RS fraction. Le et al. (2009) also concluded that the highly‐branched tapioca starch produced by a combined treatment of branching enzyme and maltogenic amylase had the potential to maintain blood glucose level after intake and during prolonged exercise. Therefore, the high amount of SDS and RS obtained from effective modification by MAse helped maintaining blood glucose bester other treatments during 120 min.

### 3.5 Impact on the glycemic index (GI) in blood

The result in Table 6 showed the changes of glycemic index (GI) on nomal and diabetic mice after every week of feeding the tested samples. An animal experiment was performed on type 2 diabetic mice to examine the effect of a modified GBR‐based flour diet. During the 4‐week experimental feeding period, the mice’ food consumption rate (g per day) showed no difference in food intake between normal and diabetic mice. Glycemic index was observed in mice groups fed with varying diets (NS: normal mice fed with nonmodified GBR flour, MA: normal mice fed with GBR flour modified, T2D‐NS: type 2 diabetic mice fed with nonmodified GBR flour, T2D‐MA: type 2 diabetic mice fed with GBR flour modified, T2D‐NS‐M: type 2 diabetic mice fed with a combination of nonmodified GBR flour and metformin, and T2D‐MA‐M: type 2 diabetic mice fed with a combination of GBR flour modified and metformin) and presented in Table 6. There was a gradual decrease in glycemic index for all groups of mice after 4 weeks feeding except for T2D‐NS group (type 2 diabetic mice fed nonmodified GBR fluor); however, there was no significant difference throughout the weeks (*p* < .05). And type 2 diabetic mice had a GI level significantly higher than normal mice (*p* < .05). The diet of modified GBR flour had the lowest level of GI in both nondiabetic and diabetic groups than the control diet (nonmodified flour). Though GI parameters slightly reduced, there was no difference between control diet and two experimental diets (modified GBR flour and nonmodified GBR flour with metformin) in diabetic mice (*p* < .05). In the diabetic mice population, on the other hand, when those fed with the modified GBR flour combined with metformin was compared to those fed with the existing diets showed a statistically significant decrease in GI level of 14.37 in the fourth week (*p* < .05). Meanwhile, the GI level of diets including nonmodified GBR flour, modified GBR flour and nonmodified GBR flour with metformin were 26.37, 22.1 and 20.1, respectively. Besides, the diabetic mice fed with modified GBR flour showed that blood glucose was maintained at the initial level. GI is an indicator of postprandial hyperglycemia of carbohydrate food and reflects the rate at which glucose is absorbed from the gastrointestinal tract into the bloodstream and cellular tissues (Gong et al., 2020; Isabel et al., 1997). The effects of a GBR diet on diabetic complications using streptozotocin‐induced diabetic rats have also evaluated (Wu et al., 2013). Hsu et al. (2008) suggested that the intake of GBR as a staple food in type 2 diabetic patients was useful in improving blood glucose levels. The GABA, tocopherols, the vitamins, minerals, and/or unknown bioactive lipids in GBR may also be associated with the effect of lowering postprandial glycemic response or preventing diabetic complications (Chou et al., 2009). Besides, Imam et al. (2012) also suggested that the benefits from GABA, oryzanol, phenolics, and other BCs in GBR may be optimal when consumed as a whole food (GBR) compared to its BCs. Another study concluded that GBR reduced plasma glucose and weight more than metformin, while white rice worsened glycemia over 4 weeks of intervention (Imam & Ismail, 2013). Furthermore, Mitra et al. (2007) reported that the high RS containing rice reduced fasting blood glucose. This may be due to the RS behaving as dietary fiber which reduces blood glucose level. The GI values of rice starches were highly positively correlated with RDS values, whereas the GI values were highly negatively correlated with RS (Pham et al., 2015). So, these results in Table 6 demonstrated that modified GBR flour was beneficial in maintaining a low glycemic index for diabetes.

**Table 6:** Changes of glycemic index (GI) on diabetic mice after every week of feeding the tested samples

Sample name	Time (week)				
	0	1	2	3	4
NS	7.67 ± 3.89^aB^	7.47 ± 3.23^aA^	7.03 ± 2.62^aA^	6.77 ± 2.09^aA^	6.57 ± 1.52^aA^
MA	7.13 ± 0.97^aB^	6.93 ± 0.85^aA^	6.80 ± 0.82^aA^	6.33 ± 1.03^aA^	6.10 ± 0.26^aA^
T2D‐NS	22.80 ± 0.26^cB^	23.80 ± 0.70^cA^	24.30 ± 0.26^cA^	24.87 ± 1.17^cA^	26.37 ± 1.50^cA^
T2D‐MA	23.63 ± 4.48^cB^	23.20 ± 1.68^cA^	22.90 ± 2.60^cA^	22.77 ± 4.51^cA^	22.10 ± 2.34^cA^
T2D‐NS‐M	32.57 ± 1.27^cB^	23.43 ± 3.91^cA^	22.60 ± 2.71^cA^	20.40 ± 3.87^cA^	20.10 ± 2.31^cA^
T2D‐MA‐M	27.20 ± 2.70^bB^	19.47 ± 2.63^bA^	17.57 ± 2.73^bA^	17.27 ± 4.82^bA^	14.37 ± 1.36^bA^

Note

The lowercase letters (a, b, c, d) in superscript indicated a statistically significant difference between treatments in each time by column (*p* < .05). The uppercase letters (A, B, C, D) in superscript indicated a statistically significant difference between the time frames of each treatment by row (*p* < .05). NS: normal mice fed nonmodified GBR flour, MA: normal mice fed GBR flour modified, T2D‐NS: type 2 diabetic mice fed nonmodified GBR flour, T2D‐MA: type 2 diabetic mice fed GBR flour modified, T2D‐NS‐M: type 2 diabetic mice fed combination nonmodified GBR flour with metformin and T2D‐MA‐M: type 2 diabetic mice fed combination GBR flour modified with metformin.

### 3.6 Biochemical analysis (Lipid profile) in blood plasma

The data in Table 7 revealed that total cholesterol, triglycerides, and LDL‐C were increased in diabetic mice. The parameters on mice that fed on modified GBR flour were lower compared to the intake of nonmodified GBR flour. The combination diet of modified GBR flour with metformin had the lowest level for the total cholesterol, triglycerides, and LDL cholesterol of 3.89, 2.06, and 0.6 mmol/L, respectively. Regarding HDL cholesterol, it was observed that plasma HDL cholesterol of diabetic mice taking modified GBR flour significantly higher by 1.94 mmol/L compared to the group taking modified GBR flour. Also, the results noticed that there was a higher level of HDL cholesterol in diabetic mice treated with metformin, especially on subjects that were maintained on metformin throughout the intervention feeding with modified GBR flour, reached the highest levels of 4.16 mmol/L and significantly different from the other groups (*p* < .05).

**Table 7:** Changes of blood plasma biochemical and antioxidant activity on diabetic mice after four week of feeding the tested samples

Sample name	Blood plasma biochemical and antioxidant activity							
	Cholesterol (mmol/L)	Triglycerides (mmol/L)	HDL‐C (mmol/L)	LDL‐C (mmol/L)	ALT (U/L)	AST (U/L)	MDA content (mM MDA/kg cells)	GSH content (mM GSH/kg cells)
NS	3.73 ± 0.26^ab^	1.45 ± 0.20^ab^	3.33 ± 0.41^b^	0.77 ± 0.16^ab^	96 ± 7.55^a^	177 ± 20.30^ab^	20.39 ± 0.99^b^	472.66 ± 16.35^b^
MA	3.20 ± 0.20^a^	1.30 ± 0.25^a^	3.52 ± 0.28^b^	0.70 ± 0.36^a^	95.67 ± 3.06^a^	140.67 ± 27.68^a^	12.41 ± 1.63^a^	626.39 ± 14.35^b^
T2D‐NS	4.56 ± 0.74^c^	3.67 ± 0.37^c^	1.38 ± 0.79^b^	1.61 ± 0.99^b^	180 ± 12^b^	292.67 ± 18.01^c^	61.15 ± 4.39^f^	79.45 ± 7.68^d^
T2D‐MA	4.45 ± 0.42^bc^	3.30 ± 0.55^c^	3.32 ± 0.63^b^	1.38 ± 0.37^ab^	137 ± 32.05^b^	239.67 ± 67.68^bc^	51.01 ± 2.07^e^	95.45 ± 12.8^c^
T2D‐NS‐M	4.25 ± 0.41^abc^	3.02 ± 0.62^b^	3.96 ± 0.55^b^	0.90 ± 0.29^ab^	121.67 ± 11.93^ab^	166.67 ± 64.29^ab^	43.98 ± 0.76^d^	227.56 ± 15.06^a^
T2D‐MA‐M	3.89 ± 0.30^bc^	2.06 ± 0.22^c^	4.16 ± 0.49^a^	0.60 ± 0.17^a^	119 ± 12.29^ab^	164.67 ± 36.36^ab^	34.96 ± 0.17^c^	247.18 ± 63.85^a^

Note

Significant differences between values in the same column were indicated by different letters (*p* < .05). NS: normal mice fed nonmodified GBR flour, MA: normal mice fed GBR flour modified, T2D‐NS: type 2 diabetic mice fed nonmodified GBR flour, T2D‐MA: type 2 diabetic mice fed GBR flour modified, T2D‐NS‐M: type 2 diabetic mice fed combination nonmodified GBR flour with metformin and T2D‐MA‐M: type 2 diabetic mice fed combination GBR flour modified with metformin.

Roohinejad et al. (2010) also demonstrated that improvement in the lipid profile of hypercholesterolemic rats after feeding with GBR for 6 weeks positively correlated with GABA content in its. Besides, Mohd‐Esa et al. (2011) reported that the consumption of a high‐fat diet supplemented with GBR was significantly reduced (lower levels of total cholesterol, LDL, LDL/HDL, malondialdehyde, and a higher level of HDL) in cardiovascular damage rabbits following 10 weeks. In the study of Imam et al. (2012), the improvements in these indices were attributed to higher amounts of GORZ, tocopherol, and monounsaturated fatty acid content. Moreover, Mitra et al. (2007) suggested that the high resistant starch (HRS) in rice reduces serum cholesterol and LDL cholesterol might be due to RS fermented to produce propionate. The positive effects of resistant starch supplementation on glucose metabolism, lipid profile, lipid peroxidation marker, and oxidative stress in overweight and obese adults are mentioned (Fereshteh et al., 2019). And, the resistant starch was also found to effectively control body weight and adipose tissue quality, while increasing the high‐density lipoprotein cholesterol (HDL‐C) concentration and lowering the glycerol, triacylglycerols (TG), total cholesterol (TC), and low‐density lipoprotein cholesterol (LDL‐C) concentrations with soybean feed (Ge et al., 2020). So, the effect on BCs and high RS to lipid profile indicated that MGBRF might also control the overweight and obese adults. Additionally, Imam et al. (2013) demonstrated that the hypocholesterolemic effects of GBR in type 2 diabetic rats are partly mediated through the upregulation of the LDL‐R and APO A1 genes. ASG, GABA, oryzanol, and phenolics from GBR contribute to the upregulation of the LDL‐R gene, and ASG, GABA, and phenolics contribute to the upregulation of the APO A1 gene. FA, whose content increased greatly after germination, had a beneficial effect in lowering the blood pressure in streptozotocin‐induced diabetic rats (Balasubashini et al., 2003). Therefore, feeding with modified GBR flour produced similar low RDS content, enhanced SDS and RS content, and increased the content of BCs, with very similar findings in results of GI, GT above, prompting the conclusion that these factors were the most important determinant of glycemia and lipid profile in the product.

### 3.7 Effect on liver function

The results of liver function in blood plasma are presented in **Table** **7**. The data showed that ALT and AST were significantly increased in diabetic mice control (T2D‐NS: type 2 diabetic mice fed with nonmodified GBR flour) by 87.5% and 65.4% relative to normal mice control (NS: normal mice fed with nonmodified GBR flour). However, all treated groups showed improvement in ALT and AST relative to diabetic mice fed with modified GBR flour, but these treated are still more than those of normal control. ALT and AST were decreased more in diabetic mice groups treated with metformin compared with others. Also, the data show that a slight decrease in ALT and AST in groups fed with modified GBR flour compared with groups fed with nonmodified GBR flour, respectively, but there were no significant differences between them (*p* < .05). According to Esa et al. (2013), the greatest reduction of ALT and AST was shown in GBR‐supplemented rabbits which suggested that antioxidant activity resulting from GBR supplementation could inhibit liver injury. Besides, Shallan et al. (2010) shown that cooked (PGBRL) ameliorated the elevation of serum lipids through decreased ALT and AST level in diabetic rats. Connecting to MDA, the changes in liver cells MDA levels of the six groups after 4 weeks are also summarized in Table 7. The basal value of liver cells MDA was significantly different in all groups of NS, MA, T2D‐NS, T2D‐MA, T2D‐NS‐M, and T2D‐MA‐*M* (*p* < .05). For the diabetic group, the levels of MDA in groups fed with modified GBR flour decreased significantly compared to nonmodified GBR flour. However, it was still greatly higher than normal mice. Moreover, this parameter occupied the lowest level in normal mice fed with nonmodified GBR flour. Regarding GSH content, it had a trend contrast to MDA content with similarity progression for all groups. It was noticed that GSH content in normal mice was significantly higher than diabetic mice. And this content was improved when fed with modified GBR flour. As a role, using metformin proved more effective and enhanced if combined with diet intake of modified GBR flour. Mohd‐Esa et al. (2011) reported that the level of MDA in plasma was significantly reduced (*p* < .05) in the group supplemented with GBR at week 10 compared to week 5. On the other hand, GORZ possesses a cholesterol‐lowering property when fed to humans and animals through decreasing cholesterol absorption and enhancing fecal sterol excretion (Sasaki et al., 1990). Besides that, Imam et al. (2014) recognized that GABA regulated hepatic cholesterol metabolism in rats when administered orally. The effects observed in the present study may also have been contributed by the synergistic effects of bioactive in GBR. Already, ASG, GABA, oryzanol, and phenolics were shown to transcriptionally affect the regulation of cholesterol metabolism and antioxidants (Imam & Ismail, 2013; Imam et al., 2013). Sergio et al. (2013) reported the beneficial effects of RS consumption in the context of an HF feeding can be driven by changes elicited at the hepatic level. The ability of the RS to correct the HF‐induced dyslipidemia and the associated IR resulted from the return to the basal expression levels of transcription factors involved in lipogenesis (SREBP‐1c), cholesterol metabolism (SREBP‐2, LXRs,) and fatty acid oxidation (PPARα). The RS feeding was able to correct the HF‐induced reduction in hepatic glucose phosphorylation and muscle glucose transport, improving glucose tolerance. Dorothy et al. (2016) reported that high amylose maize resistant starch type 2 (HAMRS2) is a fermentable dietary fiber known to alter the gut milieu, including the gut microbiota, which may explain the reported effects of resistant starch to ameliorate obesity associated metabolic dysfunction. HAMRS2 dramatically alters hepatic metabolism and gene expression concurrent with shifts in specific gut bacteria in C57BL/6J mice. Therefore, the increasing of intensive bioactive compounds together with low RDS content, high SDS, and RS content in MGBRF has positive effect on stability of biochemical function not only for glycemia and lipid profile but also for liver profile.

## 4 CONCLUSION

MAse can help to increase SDS, RS and reducing RDS in MGBRF. BCs increase in the flour under original states during modification. This increase makes the modified flour more effective on inhibition of growth of HepG2 cancer cells and shows a good ability for diabetes stabilization.

## ETHICAL APPROVAL

The authors declare that they have no known competing financial interest or personal relationships that could have appeared to influence the work reported in this paper. Written informed consent was obtained from all study participants. The animal experiments were done in this research in accordance with the guide for the care and use of laboratory under the approval of the scientific council, Institute of Biotechnology, Vietnam Academy of Science and Technology (VAST), Vietnam dated in 15th July, 2019.
